# Statistical estimation of deltoid subcutaneous fat pad thickness: implications for needle length for vaccination

**DOI:** 10.1038/s41598-022-05020-5

**Published:** 2022-01-20

**Authors:** Ronnie Sebro

**Affiliations:** 1 Department of Radiology, Mayo Clinic, 4500 San Pablo Rd S, Jacksonville, FL 32224 USA; 2 Center for Augmented Intelligence, Mayo Clinic, 4500 San Pablo Rd S, Jacksonville, FL 32224 USA

**Keywords:** Medical imaging, Public health

## Abstract

Current US Centers for Disease Control and Prevention intramuscular injection needle length guidelines for injection fo the deltoid muscle are based on weight and gender. The aims of this study are (1) to evaluate whether other biometric data (age, gender, height, weight and body mass index (BMI)) are better predictors of the thickness of the deltoid subcutaneous fat pad (DSFP) than weight and gender and (2) to evaluate the performance of the CDC weight-based needle length guidelines. This was a retrospective single center cohort study of 386 patients who underwent surveillance PET/CT between 01/01/2020 and 04/01/2021. Patient age, gender, height, weight, BMI and CT measurements of the DSFP were evaluated. DSFP was positively correlated with weight and BMI in men (r = 0.67, P < 0.001; r = 0.74, P < 0.001) and women (r = 0.69, P < 0.001; r = 0.75, P < 0.001) respectively. DSFP was negatively correlated with age in women (r =  − 0.19, P = 0.013). Age and BMI were better predictors of DSFP than weight. The best model to predict the DSFP is: $$DSFP in mm= 6.267199 -2.711159*Male + 0.228407*BMI + 0.012295*{BMI}^{2} -0.294211*BMI*Male -0.110063*Age + 0.085589*Age*Male$$ A 1-inch needle is expected to reach the deltoid in 85.3% of women less than 200 pounds, and 98.6% of men less than 260 pounds. This rate differed between genders (P < 0.001, odds ratio (OR) 0.08, 95% CI (0.02, 0.29)). A 1.5-inch needle is expected to reach the deltoid in 76.7% of women greater than 200 pounds, and 75.0% of men greater than 260 pounds. Current CDC deltoid intramuscular injection needle length guidelines result in women and obese individuals being more likely to receive subcutaneous injections. Age and BMI based guidelines for needle length selection are more accurate.

## Introduction

Vaccines are one of the most effect tools available for preventing infectious diseases. Vaccine administration requires appropriate technique, including appropriate selection of needle length to administer the vaccine in the intended location. Intramuscular vaccines including Hepatitis A, Hepatitis B, Rabies, Influenza, Pneumococcal, Diptheria toxoid and Coronavirus-19 (COVID-19), are to be administered intramuscularly to have maximum efficacy, and are usually within the deltoid muscle^1,2^. Inappropriate (too short) needle length has been shown to be associated with subcutaneous administration of the vaccine injectate^3–8^. Subcutaneous administration of injectate results in decreased vaccine efficacy^1,2^ and has been shown to lead to decreased seropositivity and decreased antibody titers compared to intramuscular injections^3–5,9^.

Several studies have tried to predict the optimal needle length for deltoid intramuscular injections^10,11^. The optimal needle length required for a deltoid intramuscular injection required estimation of the distance from the skin overlying the deltoid muscle to the deltoid muscle, which is the deltoid subcutaneous fat pad (DSFP) thickness and the distance from the skin overlying the deltoid to the humerus^10,11^. A study by Poland et al. evaluated the thickness of the deltoid subcutaneous fat pad (DSFP) in 220 healthy healthcare workers using ultrasound and showed that the DSFP varied by gender and weight, with heavier individuals having a thicker DSFP^10^. This study was the basis for the current United States Centers for Disease Control and Prevention (CDC) needle length guidelines for deltoid intramuscular injections. Current CDC guidelines recommend a needle length of 1 inch for women weighing less than 200 pounds, and for men weighing less than 260 pounds; and recommend a needle length of 1.5 inches for women weighing 200 pounds or more, and for men weighing 260 pounds or more^10^. The current CDC guidelines are based on gender and weight only. This means that the recommended needle length for a 5-foot 4-inch -tall (1.626 m) man who weighs 259 pounds (117.7 kg) with a body mass index (BMI) of 44.5 kg/m^2^ is the same as that for a 6-foot 7-inch-tall (2.01 m) man who weighs 259 pounds (117.7 kg) with a BMI of 29.2 kg/m^2^. Similarly, the CDC recommended needle length for a 5-foot-tall (1.524 m) woman who weighs 199 pounds (90.5 kg) with a BMI of 38.9 kg/m^2^ is the same as that for a 6-foot-tall (1.829 m) woman who weighs 199 pounds (90.5 kg) with a BMI of 27.0 kg/m^2^. In each of these examples, one individual is morbidly obese and the other is slightly overweight.

We hypothesized that the CDC guidelines may be inadequate for clinical practice as they rely on weight, rather than on BMI. The goal of this study is to use statistical modeling using patient clinical and demographic factors (age, gender, height, weight and BMI) to predict DSFP thickness and to better predict the optimal needle length required to achieve an intramuscular deltoid injection.

## Methods

The study experimental protocol was reviewed and approved by the local Institutional Review Board at the Mayo Clinic, Florida (IRB approval # 21-003745), and the need for signed informed consent from each patient was waived. All methods were carried out in accordance with the Health Insurance Portability and Accountability Act of 1996 (HIPAA) guidelines and regulations.

Prior reports have measured the DSFP using ultrasound and tried to avoid compression of the deltoid tissues by using “light” probe pressure or used a "stand-off pad" to increase the near field resolution of the skin line^10,11^. It is unclear how much pressure is “light” pressure and how much this “light” pressure could distort the subcutaneous fat, since by nature fat is soft and easily compressible. Ultrasound by nature of its compressive technique often distorts/compresses the subcutaneous tissues^10,11^, so ultrasound was not chosen as the modality to obtain measurements for this study. Magnetic resonance imaging (MRIs) of the shoulder were considered, however, MRIs of the shoulder utilize a shoulder coil. This shoulder coil also potentially compresses the subcutaneous tissues, and so MRIs were not chosen as the modality to obtain measurements. Chest computed tomography (CTs) were considered as these studies often include the shoulder, however, the arms are generally held above the head to minimize artifacts and radiation dose to the patient, so chest CTs were not utilized. The CT component of whole-body positron emission tomography/computed tomography (PET/CTs) often includes the shoulder in the anatomic position and generally have no mass effect on the subcutaneous tissues because the patient is lying supine, so this modality was chosen to obtain measurements.

### PET/CT technique

The CT component of PET/CTs of 1000 consecutive patients imaged at a tertiary care academic center between 01/01/2020 and 04/01/2021 were retrospectively reviewed. PET/CT scans were performed using a Siemens Biograph PET/CT scanner with a 128-slice CT scanner (Siemens Healthineers, Malvern, PA, USA) using a slice thickness of 4 mm, field of view (FOV) of 500 mm, pitch of 0.8, 120 kVp, and tilt of 0°. 2-Deoxy-2-[^18^F] fluoroglucose (^18^F-FDG) is a standard radiotracer used for PET/CT imaging and ^18^F-FDG was injected intravenously into patients 60 min prior to each PET/CT scan. Measurements were taken to the nearest 0.1 mm using the Visage 7 Picture Archiving and Communications Systems (PACS) (San Diego, CA, USA) measurement tool. Patients were excluded if their arms were positioned over the head or if they had a shoulder arthroplasty (n = 614). Clinical and demographic variables including age, gender, height, weight and BMI were recorded at the time of the PET/CT scan.

### DSFP measurement technique

**Figure 1:**
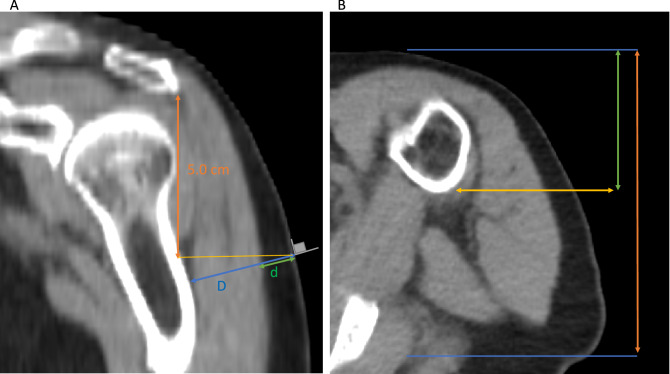
(**A**) Coronal CT image of a 77-year-old man with history of head and neck squamous cell carcinoma, weight of 74 kg, height of 1.70 m, demonstrating the deltoid subcutaneous fat pad (DSFP) measurement technique. Orange arrow identifies the region exactly 5.0 cm inferior to the acromion. Yellow line is perpendicular to the orange line and identifies the estimated injection site. 90° angle at the estimated injection site in gray was used to identify the trajectory of the needle. Green arrow identifies the distance from the skin surface to the deltoid muscle measured perpendicular to the skin in the coronal plane (note arrow is jittered to show blue arrow along the same path) (DSFP = d = 11.2 mm). Blue arrow identifies the distance from the skin surface to the humerus measured perpendicular to the skin in the coronal plane (D = 40.3 mm). (**B**) Axial CT image of the same man demonstrating identification of the correct coronal plane to measure the DSFP. Blue lines identify the anterior and posterior borders of the shoulder 5.0 cm inferior to the acromion. The orange arrow measures the distance between the anterior and posterior borders of the shoulder. The green arrow measures the half-way distance between the anterior and posterior borders of the shoulder. The yellow arrow was used to identify the coronal plane to take measurements of the deltoid subcutaneous fat pad.

A fellowship-trained musculoskeletal radiologist with over 12 years of experience evaluated scans and made measurements on the left arm in all but two patients who had intramuscular lipomas in the deltoid. The left arm was chosen for measurements because it is the common non-dominant arm and usually chosen for deltoid injections. The distance from the skin overlying the deltoid to the deltoid muscle, d (equivalent to the DSFP); and the distance from the skin overlying the deltoid to the humerus, D, were measured at exactly 5 cm inferior to the acromion in the mid anterior–posterior shoulder at 90° perpendicular to the skin measured in the coronal plane, following the CDC guidelines^12^ (Fig. 1). Two millimeters of the needle was assumed to be above the skin to facilitate retrieval of the needle in the event of a fractured needle. A previous manuscript noted that an injection would be considered intramuscular if the needle would penetrate the deltoid muscle by at least 5 mm^10^, so this threshold was used.

### Statistical analyses

T-tests were used to compare mean weights and mean DSFP thickness for men and women. Pearson’s correlation coefficients were used to evaluate the correlations between DSFP thickness and weight, BMI, age and height for men and women. Receiver operator characteristic (ROC) curves were used to evaluate the predictive performance of age, height, weight, and BMI to predict whether injections were intramuscular versus subcutaneous using the CDC current guidelines. DeLong’s test was used to compare ROC curves. Next, logistic regression was used to predict the odds of an inadvertent subcutaneous model using the CDC guidelines, comparing patients above the CDC weight threshold (200 pounds for women, 260 pounds for men) compared to patients below the CDC weight threshold.

Predictive statistical modeling was used to estimate a patient’s DSFP based on age, gender, height, weight, and BMI using generalized linear models. The Shapiro-Wilks test was performed to confirm the distribution of DSFP was not significantly different from a normal distribution. First, univariate linear regression models to predict DSFP using age, height, weight and BMI as covariates were created. Age (P < 0.001), height (P < 0.001), weight (P < 0.001) and BMI(P < 0.001) were significant univariate predictors of DSFP.

We built the best multivariable model to predict DSFP using age, gender, height, and weight (model 1). Variables with P-values < 0.20 in the univariate analysis were included in the first multivariable model, and we removed terms with P-values ≥ 0.05 in this multivariable model using stepwise backward regression. We subsequently included all squared terms for each remaining variable to the multivariable model. Variables which were not statistically significant (P ≥ 0.05) were removed from the model using stepwise backward regression. Next, all pairwise interaction terms between the remaining variables in the multivariable model were added to the multivariable model. Variables which were not statistically significant (P ≥ 0.05) were removed from the model using stepwise backward regression. The final model 1 contained the variables age, gender, weight, age*gender, age*weight and gender*weight (Adjusted R-squared = 0.565, Akaike Information Criterion (AIC) = 2289.8). Full details are shown in Supplementary material 1.

We built the best multivariable model to predict DSFP using age, gender, height, and BMI (model 2). Variables with P-values < 0.20 in the univariate analysis were included in the first multivariable model, and we removed terms with P-values ≥ 0.05 in this multivariable model using stepwise backward regression. We subsequently included all squared terms for each remaining variable to the multivariable model. Variables which were not statistically significant (P ≥ 0.05) were removed from the model using stepwise backward regression. Next, all pairwise interaction terms between the remaining variables in the multivariable model were added to the multivariable model. Variables which were not statistically significant (P ≥ 0.05) were removed from the model using stepwise backward regression. The final model 2 included the following variables: age, gender, BMI, BMI-squared, gender*BMI interaction term, age and age*gender interaction term (Adjusted R-squared = 0.648, AIC = 2204.1). Model 2 was compared to model 1 using the Akaike Information Criterion (AIC). Model 2 was superior to model 1 and was used for predictive modeling.

The best model to predict the DSFP is:

1 $$DSFP\,in\, mm= 6.267199 -2.711159*Male + 0.228407*BMI + 0.012295*{BMI}^{2} -0.294211*BMI*Male -0.110063*Age + 0.085589*Age*Male$$

Model 2 (Eq. 1) was used to create plots of the predicted thickness of the DSFP in men and women aged 35 and 75 years with varying BMIs (Figure iv). Statistical analyses were conducted using *Rv4.04*. All tests were two-sided and P-values less than 0.05 were considered statistically significant.

## Results

386 patients with a median age of 68 years (range 19 to 93 years old) were evaluated. The other 614 patients were excluded because their arms were positioned over the head during imaging. Most patients (96.9%) underwent PET/CT for surveillance of malignancy. Patients’ demographics are shown in Table 1. Although men were on average heavier than women (P < 0.001, 95% CI (9.2, 16.3) kg), women had thicker DSFPs than men (P < 0.001, 95% CI (4.4, 7.3) mm). DSFP was positively correlated with weight in men (r = 0.67, P < 0.001) and women (r = 0.69, P < 0.001); and with BMI in men (r = 0.74, P < 0.001) and women (r = 0.75, P < 0.001). DSFP was negatively correlated with age in women (r =  − 0.19, P = 0.013) and borderline negatively correlated with age in men (r =  − 0.13, P = 0.054). We found no correlation between DSFP and height in either men (r =  − 0.04, P = 0.601) or women (r =  − 0.03, P = 0.691).

**Table 1:** Patient clinical and demographic characteristics.

Variable	All	Male (N = 219)	Female (N = 167)	P-value
Mean age in years (range)	67.5 (19–93)	68.7 (21–90)	65.9 (19–93)	0.027^†^
**Race/ethnicity**				
American Indian/Alaska Native (AINA)	1 (0.3%)	0 (0.0%)	1 (0.6%)	0.388^§^
Asian	7 (1.8%)	3 (1.4%)	4 (2.4%)	
Black	28 (7.3%)	13 (5.9%)	15 (9.0%)	
Hispanic	10 (2.6%)	7 (3.2%)	3 (1.8%)	
Native Hawaiian/Pacific Islander (NHPI)	1 (0.3%)	0 (0.0%)	1 (0.6%)	
Other	5 (1.3%)	4 (1.8%)	1 (0.6%)	
White	334 (86.5%)	192 (87.7%)	142 (85.0%)	
History of cancer	374 (96.9%)	213 (97.3%)	161 (96.4%)	0.769^§^
Mean height in m (SD)	1.71 (0.1)	1.77 (0.07)	1.63 (0.07)	< 0.001^†^
Mean weight in kg (SD)*	80.9 (18.6)	86.4 (17.5)	73.7 (17.5)	< 0.001^†^
Mean BMI in kg/m^2^ (SD)*	27.6 (5.8)	27.4 (5.3)	27.8 (6.4)	0.484^†^
Mean deltoid subcutaneous fat pad, d in mm (SD)	12.2 (7.2)	9.6 (4.9)	15.5 (8.3)	< 0.001^†^
Mean deltoid thickness from skin to bone, D in mm (SD)	37.2 (12.4)	35.6 (10.8)	39.4 (14.1)	0.004^†^

*One man and one woman did not have recorded weights at imaging.

^†^By 2-sample t-test with unequal variances.

^§^By Fisher’s exact test.

**Figure 2:**
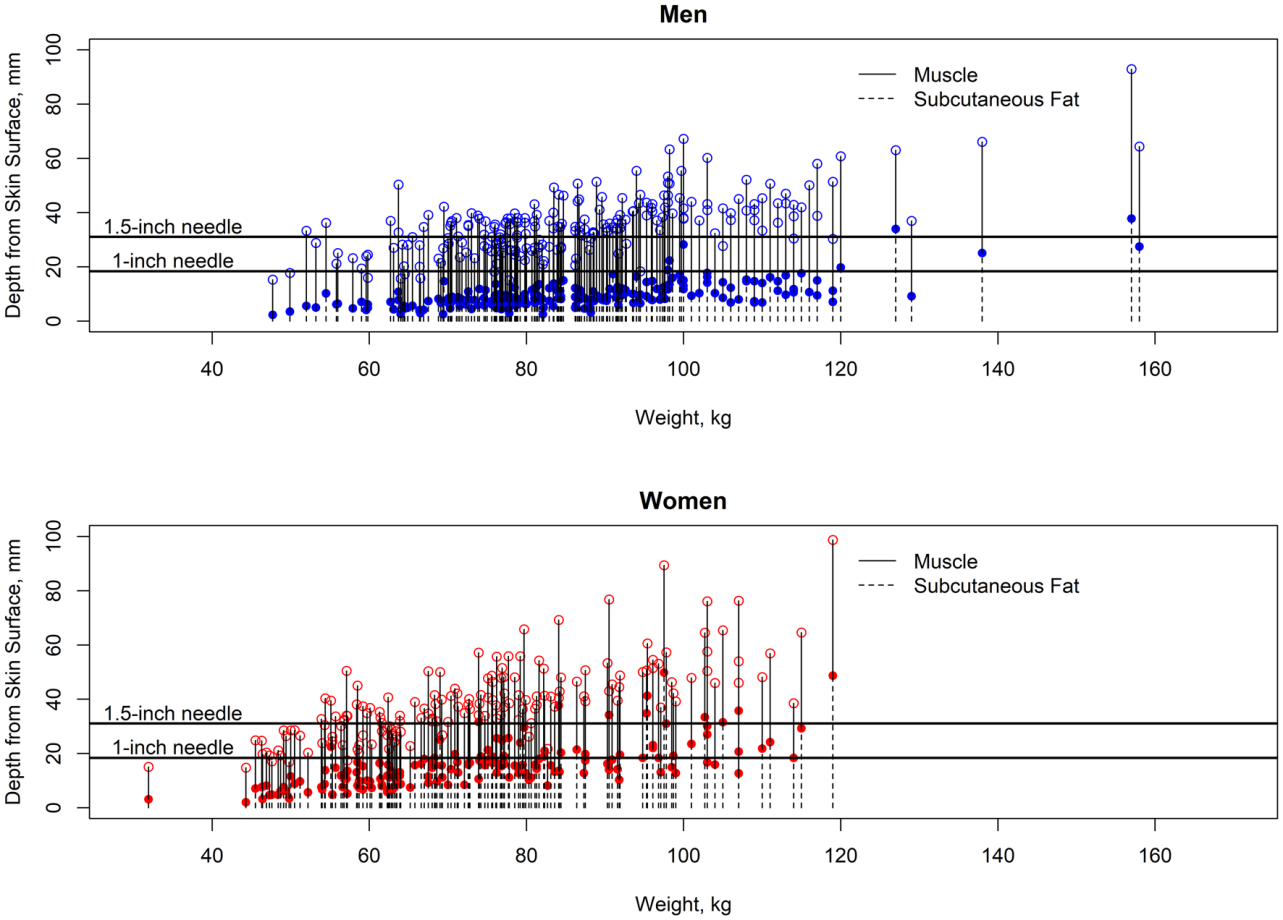
Plots of the deltoid subcutaneous fat and muscle thickness in men and women versus weight in kg. Weight in kilograms for each male (top) and female (bottom) individual, with CT measurements of depth from skin surface (0 mm) to bone. The skin-to-muscle distance (which defines the deltoid subcutanous fat pad thickness) is represented by dotted lines ending in a solid circle, while the muscle-to-bone distances (which defined muscle thickness) is represented by solid lines ending in an open square. Bone is represented by the area above the open circles. Blue filled circles – Distance from skin surface to the deltoid muscle surface in men. Blue unfilled circles – Distance from the skin surface to the humerus in men. Red filled circles – Distance from skin surface to the deltoid muscle surface in women. Red unfilled circles – Distance from the skin surface to the humerus in women. Horizontal black lines represent the effective maximum penetration depth of 1-inch and 1.5-inch needles.

**Figure 3:**
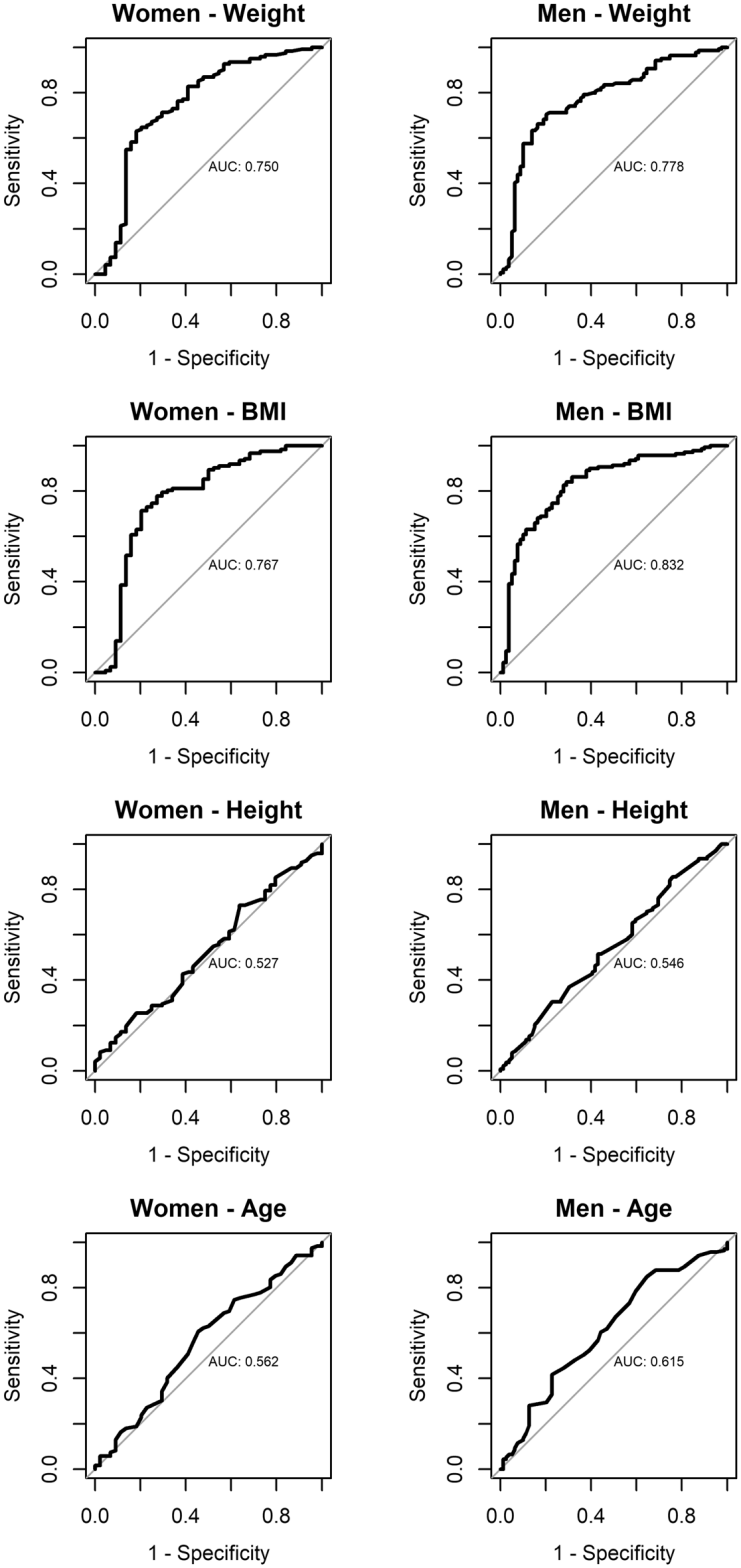
Receiver operator curves evaluating weight, BMI, height and age to predict intramuscular injections using the CDC guidelines (1-inch needle for females less than 200 pounds; 1.5-inch needle for females greater than 200 pounds; 1-inch needle for males less than 260 pounds; 1.5-inch needle for males greater than 260 pounds).

Our results show that a 1-inch needle is expected to reach the deltoid in 85.3% (116/136) of women less than 200 pounds, and 98.6% (207/210) of men less than 260 pounds. This rate differed between genders (P < 0.001, odds ratio (OR) 0.08, 95% CI (0.02, 0.29)). A 1.5-inch needle is expected to reach the deltoid in 76.7% (23/30) of women greater than 200 pounds, and 75.0% (6/8) of men greater than 260 pounds (Fig. 2). The odds of inadvertent subcutaneous injection in men greater than 260 pounds and women greater than 200 pounds (above the CDC weight thresholds) are over 4 times higher than those below the threshold (OR = 4.33, P = 0.002) when using the CDC-recommended needle lengths. BMI was the best predictor of DSFP in men but was not significantly better than weight as a predictor in women (Fig. 3, Supplementary Fig. 1). For women, the area under the receiver-operator curve (ROC) curve (AUC) for BMI (0.767) to predict an intramuscular injection was significantly better than the AUC for height (AUC = 0.527, P < 0.001) and the AUC for age (AUC = 0.562, P < 0.001) but was not significantly different from the AUC for weight (AUC = 0.750, P = 0.210). For men, we found that the AUC for BMI (AUC = 0.832) to predict an intramuscular injection was significantly better than the AUC for weight (AUC = 0.777, P = 0.001), the AUC for height (AUC = 0.546, P < 0.001) and the AUC for age (AUC = 0.612, P < 0.001).

The best model to predict the DSFP is:

2 $$DSFP\, in\, {{mm}} = 6.267199 -2.711159*Male + 0.228407*BMI + 0.012295*{BMI}^{2} -0.294211*BMI*Male -0.110063*Age + 0.085589*Age*Male$$

**Figure 4:**
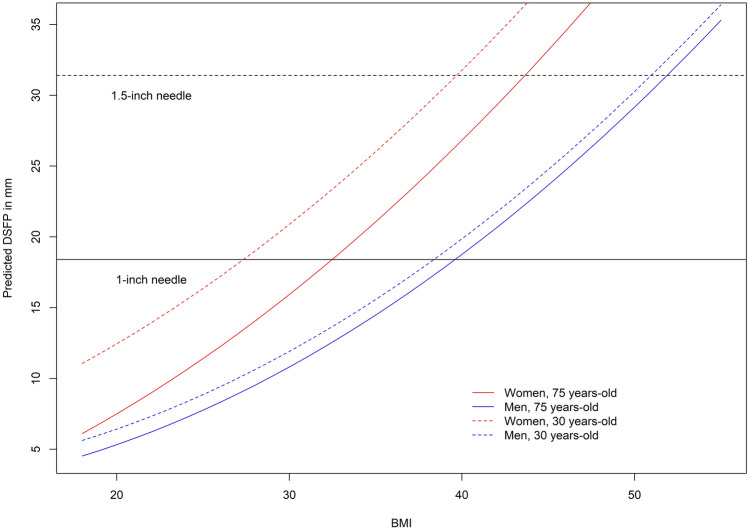
Predictive modeling of DSFP thickness in mm by BMI for individuals from Eq. 1. Red solid line represents the predicted DSFP for women aged 75 years. Red broken line represents the predicted DSFP for women aged 30 years. Blue solid line represents the predicted DSFP for men aged 75 years. Blue broken line represents the predicted DSFP for men aged 30 years.

We used this model to predict the DSFP thickness for males (aged 30 and 75 years) and females (aged 30 and 75 years) with varying BMI (Fig. 4).

## Discussion

In this study, we show that both weight and BMI were strongly correlated with DSFP in men and women. Despite weighing less on average than men, women tended to have thicker DSFPs than men. In our predictive modeling, we found that age, gender and BMI were more predictive of DSFP than the best model using age, gender and weight.

A deltoid intramuscular injection has been defined as an injection with penetration of the muscle by 5 mm or more, with 2 mm of needle superficial to the skin to aid in needle retrieval in the event of an accidental needle break^10^. Based on this, we conclude that the DFSP can be predicted using the following equation:

$$DSFP\, in\, {mm}= 6.267199 -2.711159*Male + 0.228407*BMI + 0.012295*{BMI}^{2} -0.294211*BMI*Male -0.110063*Age + 0.085589*Age*Male$$

The predicted DSFP can then be used to find the appropriate needle length where the appropriate needle length is predicted DSFP + 7 mm. Therefore, if the predicted DSFP < 18.4 mm, then a 1-inch (25.4 mm) needle would be appropriate. Similarly, if the predicted DSFP > 18.4 mm but < 31.1 mm, then a 1.5-inch (38.1 mm) needle would be appropriate. Because of this, a 1-inch needle may be too short to reliably get to the deltoid muscle in women, particularly in heavier women and a 1.5-inch needle may be too short to achieve an intramuscular injection in women greater than 200 pounds, and men greater than 260 pounds. Age and BMI together are better than weight for predicting DSFP thickness. DSFP decreases with age faster in women.

More recently new vaccines have been developed for COVID-19 in the wake of the COVID-19 pandemic. Currently, there is a global effort to vaccinate individuals with these new COVID-19 vaccines. This effort requires appropriate needle length selection for vaccination administration. Our results suggest that women, and in particular younger and obese women would be more likely to have inadvertent subcutaneous injections and be at increased risk for vaccine failures. Recent CDC Morbidity and Mortality Weekly Report (MMWR) data show that 63% of COVID-19 vaccine failures have been in women^13^, however, the relative proportions of males to females in the vaccinated population is unknown.

While this study and other studies have shown associations between the DSFP and weight and BMI^10,11^, there are a few differences between this study and previously published reports. The prior report by Poland et al. investigated healthcare workers aged 18–59 years, weight range of 59–118 kg and BMI of 17.1–49.9 kg/m^2^ whereas this study investigated patients aged 19–93 years, weight range of 31.9–158 kg and BMI of 13.3–55.9 kg/m^2^. This study has individuals over a greater age range, weight range and BMI range than the study by Poland et al.^10^. Similarly, Cook et al.^11^ conclude that a 1-inch needle (25.4 mm) is an appropriate length for men of all BMI, whereas this study showed that this needle length was appropriate for 96.8% of men of all BMI (211/218) and only appropriate for 37.5% (3/8) men who weighed more than 260 pounds. This is likely because our study had larger weight and BMI ranges than these prior studies. Poland et al. suggest that a 5/8 inch, 1-inch (25.4 mm) and 1.5-inch (38.1 mm) needle is appropriate for women less than 60 kg, 60–90 kg and greater than 90 kg respectively, which contradicts the finding by Cook et al. who suggested that a 1-inch needle is appropriate for all women with BMI < 35^11^. Our data suggest that the appropriate needle length for a woman is more complicated and depends on her age and BMI.

This study utilizes CT rather than ultrasound for measuring DSFP. Prior reports used ultrasound to measure the DSFP^10,11^. The advantage of using ultrasound is that it does not involve radiation, and it can be used prospectively to evaluate patients. However, ultrasound compresses the subcutaneous tissues^10,11^ and in our experience this compression likely results in underestimation of the DSFP. The advantage of using CT is that the DFSP measurement is unaffected by compressive forces on the subcutaneous tissues^6–8^, however one disadvantage of CT is that the radiation would make it difficult to prospectively evaluate healthy individuals.

One interesting finding that we have noted is that there seem to be differences in the adiposity patterns between males and females. The prior study by Cook et al.^11^ noted that females with the same BMI as males had significantly thicker subcutaneous layers (P < 0.001) and thinner muscle layers (P < 0.001). Poland et al. also noted on average thicker DSFPs in women compared to men^10^. Our analysis confirms these findings. We also find that there was a statistically significant interaction between age and gender, with the DSFP decreasing at a faster rate in women than men with aging. Prior studies have shown that with age, there is increasing internal adiposity resulting in an increase in BMI, while simultaneously a decreasing in subcutaneous fat^14^. This change is thought to be related to adipose tissue shifts from primarily subcutaneous depots to visceral depots, which are more closely associated with the development of metabolic syndrome/insulin resistance^14^. This redistribution of adipose tissue also results in sunken cheeks, prominence of wrinkles and thinning of the skin over the hands and legs due to subcutaneous fat loss^14^. Further research is required to understand the differences in whole body adiposity patterns between males and females, and to understand how these differences change with age.

This study has a few limitations. One limitation is the study sample size; however, this study is 75% larger than the study used in creating the CDC needle length guidelines^10^. There are several additional factors that may contribute to vaccine failure, including an individual’s genetic makeup, pathogen genetic variants (viral and bacterial genetic variants), injection technique including education and training of personnel who administer vaccines, and injection location. This study used the injection guidelines proposed by the CDC, which may or may not be adhered to in clinical practice. Another limitation of this study is that most of the patients underwent PET/CT for surveillance imaging for malignancy. It is unclear how a history of malignancy could affect the DSFP. However, our study population is an important population because most early deaths related to COVID-19 in the United States were related to patients with medical comorbidities including cancer and obesity^15^. Finally, most of the patients in our study were White individuals of European ancestry. It is unclear whether these results port to members of all races/ethnicities equally.

In summary, current CDC guidelines for deltoid intramuscular injections may result in subcutaneous injection and potentially decreased vaccine efficacy in women and obese individuals.

## Supplementary Information

Supplementary Appendix.

Supplementary Figure 1.
