# Supplementary material for: Statistical estimation of deltoid subcutaneous fat pad thickness: implications for needle length for vaccination
Source: Sci Rep. 2022 Jan 20;12:1069. doi: 10.1038/s41598-022-05020-5 (PMC8776900; doi:10.1038/s41598-022-05020-5)
Supplement: Supplementary file 1 — Supplementary Appendix. [file 41598_2022_5020_MOESM1_ESM.docx]

**Appendix 1**

Predictive statistical modeling was used to estimate a patient’s DSFP based on age, gender, height, weight and BMI using generalized linear models. DSFP was checked for normality using the Shapiro-Wilks test. Generalized linear regression modelling was used to predict the DSFP.

We tried to find the best regression model to predict DSFP using age, gender, height and weight, which we termed Model 1. Univariate linear regression models were used to predict DSFP using age, gender, height, and weight. Age (P<0.001), gender (P<0.001), height (P<0.001), and weight (P<0.001) were each significant predictors of DSFP. Next, a multivariable linear regression model was used to predict DSFP adjusting for all variables with P<0.20 in the univariate analysis (age, gender, height, and weight). We found that all variables (age, gender, height, and weight) were statistically significant (P<0.001 for all) in the first multivariable model. Next, we evaluated whether terms in age-squared, height-squared and weight-squared were significant predictors of the DSFP, so we added these terms to the first multivariable model to create a second multivariable model. We used stepwise backward regression to eliminate variables with P-value ≥ 0.05. We found that age-squared (P=0.747), height-squared (P=0.590) and weight-squared (P=0.671) were not statistically significant predictors of DSFP, so these terms were removed from the model. The multivariable model at this stage included age, gender and weight variables. Next, we tested for interactions between the age, gender and weight variables. We found that the interaction terms between age*gender (P=0.013), age*weight (P=0.010) and gender*weight (P<0.001) were statistically significant. The model with age, gender, weight, age*gender, age*weight and gender*weight, was termed model 1 (Adjusted R-squared=0.565, AIC=2289.8).

**Model 1:**

| **Variable** | **Estimate** | **Standard Error** | **T-value** | **P-value** |
| --- | --- | --- | --- | --- |
| Intercept | **–18.701** | **6.810** | **-2.75** | **0.006** |
| Age | 0.153 | 0.097 | 1.57 | 0.117 |
| Male Gender | -5.214 | 3.905 | -1.34 | 0.183 |
| Weight | 0.545 | 0.089 | 6.12 | <0.001 |
| Age*Male Gender | 0.110 | 0.044 | 2.49 | 0.013 |
| Age*Weight | -0.003 | 0.001 | -2.59 | 0.010 |
| Male Gender*Weight | -0.137 | 0.028 | -4.89 | <0.001 |

BMI was a significant predictor of DSFP in the univariate linear regression model (P<0.001). We tried to find the best multivariable regression model to predict DSFP using age, gender, height and BMI. Age (P<0.001), gender (P<0.001) and BMI (P<0.001) were statistically significant predictors of DSFP in this first multivariable model, however height (P=0.659) was not significant, so the variable height was removed from the model. Next, we evaluated whether terms in age-squared, and BMI-squared were significant predictors of the DSFP, so we added these terms to the multivariable model to create a second multivariable model. We used stepwise backward regression to eliminate variables with P-value ≥ 0.05. We found that age-squared (P=0.868) was not statistically significant, so it was removed from the model, whereas the variable BMI-squared (P=0.011) was retained in the multivariable model.

The multivariable model at this stage included age, gender, BMI, and BMI-squared variables. Next, we tested for pair-wise interactions between the age, gender, BMI, and BMI-squared variables. We used a likelihood ratio test to evaluate whether any of the interaction terms with BMI-squared (age*BMI-squared, gender*BMI-squared, BMI-cubed) were statistically significant. The likelihood ratio test showed that none of these interaction terms with BMI-squared were statistically significant (F=1.86, 3 df, 375 df, P=0.136). These interaction terms with BMI-squared were removed from the model. There was no significant interaction between age and BMI (P=0.155), so this interaction term was removed from the model. The final model included age, gender, BMI, BMI-squared, gender*BMI interaction term, and age*gender interaction term and was termed model 2 (Adjusted R-squared=0.648, AIC=2204.1).

**Model 2:**

| **Variable** | **Estimate** | **Standard Error** | **T-value** | **P-value** |
| --- | --- | --- | --- | --- |
| Intercept | **6.267** | **4.02** | **1.56** | **0.120** |
| Age | -0.110 | 0.03 | -4.19 | <0.001 |
| Male Gender | -2.711 | 3.39 | -0.80 | 0.424 |
| BMI | 0.228 | 0.24 | 0.97 | 0.333 |
| BMI-squared | 0.012 | 0.004 | 3.17 | 0.002 |
| Age*Male Gender | 0.086 | 0.04 | 2.30 | 0.022 |
| Male Gender*BMI | -0.294 | 0.076 | -3.88 | <0.001 |

We compared model 1 to model 2 using the Akaike Information Criterion (AIC) and found that the model 2 (R-squared 0.648, AIC 2204.1) performed better than model 1 (R-squared 0.565, AIC 2289.8).
