# Supplementary figures and images for: Statistical estimation of deltoid subcutaneous fat pad thickness: implications for needle length for vaccination
Source: Sci Rep. 2022 Jan 20;12:1069. doi: 10.1038/s41598-022-05020-5 (PMC8776900; doi:10.1038/s41598-022-05020-5)

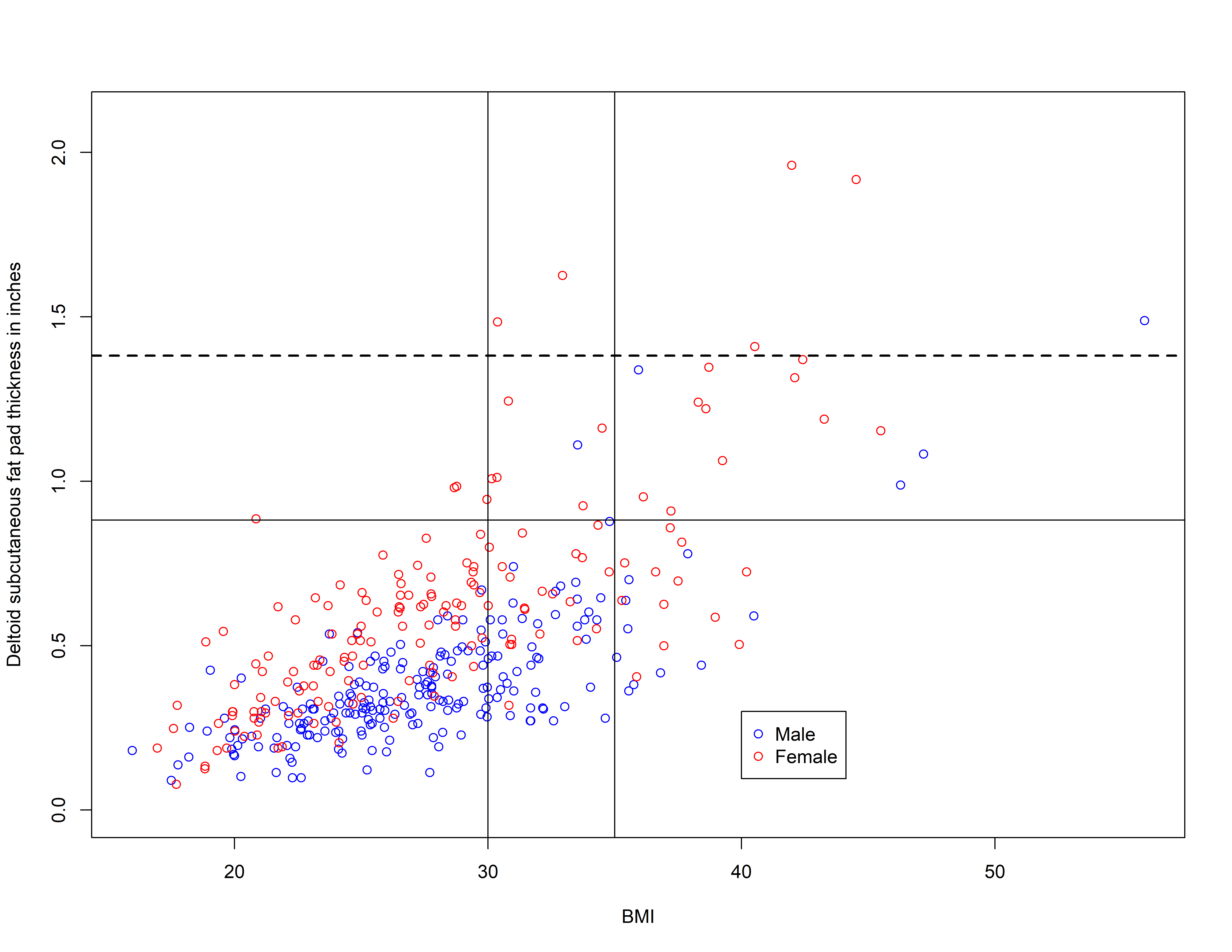

Supplement: Supplementary file 2 — Supplementary Figure 1. [file 41598_2022_5020_MOESM2_ESM.tiff]
